# Clinical pharmacist-led program on medication reconciliation implementation at hospital admission: experience of a single university hospital in Croatia

**DOI:** 10.3325/cmj.2016.57.572

**Published:** 2016-12

**Authors:** Ivana Marinović, Srećko Marušić, Iva Mucalo, Jasna Mesarić, Vesna Bačić Vrca

**Affiliations:** 1 Hospital Pharmacy, University Hospital Dubrava, Zagreb, Croatia; 2 Department of Clinical Pharmacology, University Hospital Dubrava, Zagreb, Croatia; 3 Faculty of Pharmacy and Biochemistry, University of Zagreb, Zagreb, Croatia; 4 Agency for Quality and Accreditation in Health Care and Social Welfare, Zagreb, Croatia

## Abstract

**Aim:**

To evaluate the clinical pharmacist-led medication reconciliation process in clinical practice by quantifying and analyzing unintentional medication discrepancies at hospital admission.

**Methods:**

An observational prospective study was conducted at the Clinical Department of Internal Medicine, University Hospital Dubrava, during a 1-year period (October 2014 – September 2015) as a part of the implementation of Safe Clinical Practice, Medication Reconciliation of the European Network for Patient Safety and Quality of Care Joint Action (PASQ JA) project. Patients older than 18 years taking at least one regular prescription medication were eligible for inclusion. Discrepancies between pharmacists' Best Possible Medication History (BPMH) and physicians' admission orders were detected and communicated directly to the physicians to clarify whether the observed changes in therapy were intentional or unintentional. All discrepancies were discussed by an expert panel and classified according to their potential to cause harm.

**Results:**

In 411 patients included in the study, 1200 medication discrepancies were identified, with 202 (16.8%) being unintentional. One or more unintentional medication discrepancy was found in 148 (35%) patients. The most frequent type of unintentional medication discrepancy was drug omission (63.9%) followed by an incorrect dose (24.2%). More than half (59.9%) of the identified unintentional medication discrepancies had the potential to cause moderate to severe discomfort or clinical deterioration in the patient.

**Conclusion:**

Around 60% of medication errors were assessed as having the potential to threaten the patient safety. Clinical pharmacist-led medication reconciliation was shown to be an important tool in detecting medication discrepancies and preventing adverse patient outcomes. This standardized medication reconciliation process may be widely applicable to other health care organizations and clinical settings.

Medication reconciliation is designated as “the process of identifying the most accurate list of a patient's current medicines including the name, dosage, frequency, route and comparing them to the current list in use, recognizing any discrepancies, and documenting any changes, thus resulting in a complete list of medications, accurately communicated” (1). This definition described by the Institute for Healthcare Improvement highlights the need for accuracy in obtaining the Best Possible Medication History (BPMH), ie, a comprehensive list of all home medications taken by the patient prior to hospital admission (2). Moreover, if the goal is to prevent inappropriate or interrupted drug therapy during hospitalization, the most accurate and complete BPMH needs to be recorded at the time of admission and appropriately transferred in the process of care.

Review of the literature disclosed ample evidence showing the occurrence of medication discrepancies between the BPMH and physicians’ admission medication orders and revealed a high incidence of medication discrepancies (3-9). According to the systematic literature review, 27%-54% of the patients had at least one medication history discrepancy at hospital admission, with 19%-75% of these being unintentional (10), thus carrying a huge potential to adversely affect patient safety (3,4,6,8). Many studies have demonstrated the positive effect of medication reconciliation on identifying and rectifying medication errors at the time of admission (3,11-15). Most of them involved pharmacists, who have been shown to be more thorough than other health care professionals in obtaining a complete medication history (16-18). A recent meta-analysis confirmed that pharmacy-led medication reconciliation interventions were an effective strategy in reducing medication discrepancies and had a greater impact when conducted at either admission or discharge (19). Moreover, such interventions were proven to be cost-effective (20,21).

The process of medication reconciliation has been advocated by many patient safety organizations and authorities trying to facilitate its implementation (22-25). In 2006, the World Health Organization (WHO) launched the High 5s Project with an aim of reducing patient safety problems by implementing and evaluating Standard Operating Procedures (SOPs) across countries (24). “Medication Accuracy at Transitions in Care”, or Medication Reconciliation, was one of the fully developed SOPs. It was considered important to determine the feasibility of its implementation and to measure the impact on patient safety in different health care environments. To help establish consistency in a range of health care systems, the WHO High 5s Protocol on Medication Reconciliation and Implementation Guide was published in 2015 (26).

The implementation of medication reconciliation in Croatia has been initiated within the scope of the Work Package 5 of the European Union Network for Patient Safety and Quality of Care (PaSQ) project (27), which was adapted from the original WHO High 5s Protocol on Medication Reconciliation and Implementation Guide. The goal of this project was to initiate the implementation of safe clinical practice, including medication reconciliation, across Croatian hospitals and to collect and report the data on its impact on patient safety. Since 2014, University Hospital Dubrava, Zagreb, has been participating in the implementation of medication reconciliation project activities supported by the Croatian PaSQ National Contact Point (NCP), Agency for Quality and Accreditation in Healthcare and Social Welfare.

The primary aim of this study was to evaluate the process of clinical pharmacist-led medication reconciliation implementation in clinical practice by quantifying and analyzing unintentional medication discrepancies at hospital admission. The secondary aim was to investigate possible patient-related determinants influencing medication discrepancies.

## Participants and methods

### Setting and participants

This prospective, observational study was conducted at the Clinical Department of Internal Medicine, University Hospital Dubrava, Zagreb, a 600-bed teaching hospital during a 1-year period from October 2014 to September 2015. All patients included in the study were randomly selected, using a computer-generated random number table, upon admission to the hospital after the routine primary medication history had been taken. Patients were considered eligible for the study if they were aged 18 years and older and taking at least one regular prescription medication. Patients were excluded if they were not able to answer the questions needed to complete the structured interview, did not have a caregiver who could be interviewed in case the patient was unable to participate in the interview, were unable or unwilling to give their consent, or were transferred from another ward. Ethical approval of the study was obtained from the Hospital Ethics Committee and all patients gave their written informed consent before taking part in the study.

### Medication reconciliation process

Medication reconciliation process was based on the WHO Standard Operating Protocol and Implementation Guide adapted from the original WHO High 5s Protocol on Medication Reconciliation and Implementation Guide (26). To standardize the process of data collection and medication reconciliation, two clinical pharmacists, formally trained by the PASQ Croatia NCP, conducted the medication reconciliation process within 24 hours following hospital admission. A patient interview was undertaken by using a standardized BPMH form. A thorough history of all regular preadmission medication use (prescription, over-the-counter, dietary supplements, vitamins, herbal preparations, parenteral nutrition) was taken and completed using other sources of information including previous hospital records (discharge summaries), examination of the medication vials, review of a home medication list, caregiver interview, and/or communication with outpatient pharmacy and/or general practitioner (GP). All available sources of information used to obtain the BPMH were recorded. Furthermore, the BPMH included the information about relevant demographic and clinical data, experienced adverse drug events, social history, and understanding of preadmission medications and level of adherence. Patients’ level of understanding of their preadmission therapy was assessed as high, medium or low, depending on whether the patient could provide the name, dose, route, and frequency of the medication use and its intended indication (5). The BPMH was then compared to admission orders prescribed by physicians to detect possible medication discrepancies. To improve patient safety and prevent medication errors from occurring during the health care process, pharmacists communicated directly with physicians in order to clarify whether the observed therapy changes were intentional or unintentional.

### Classification of unintentional medication discrepancies

Unintentional medication discrepancies without any clinical rationale were considered medication errors (5) and were classified according to the type of discrepancy into one of following categories: drug omission or addition, substitution of a medication within the same pharmacological group, incorrect dose, incorrect frequency, or incorrect route of administration. All unintentional medication discrepancies were presented to the expert panel team consisting of a clinical pharmacist and a clinical pharmacologist. Medication discrepancies were discussed and classified by their potential to cause harm, according to the method developed by Cornish et al (3). Additional sources of patient information, such as previous discharge summaries and laboratory test results, were reviewed as needed. All disagreements were resolved and consensus was achieved. The degree of impact of each medication discrepancy was defined as follows: a) class 1 – discrepancies unlikely to cause patient discomfort or clinical deterioration, b) class 2 – discrepancies with the potential to cause moderate discomfort or clinical deterioration, and c) class 3 – discrepancies with the potential to result in severe discomfort or clinical deterioration (3).

### Outcome measures and data collection

The primary outcome measure included the frequency, type, and potential severity of unintentional medication discrepancy derived by comparison of physicians’ admission medication orders and BPMH obtained by clinical pharmacists. To identify the predictors of unintentional medication discrepancies, the data collected in the present study were patient demographic factors (age, sex, educational level, employment status, residence, type of hospital admission); clinical characteristics (comorbidities, readmission medication, history of adverse drug events, recent hospitalization, length of hospital stay); and level of patients' understanding of preadmission medication.

### Statistical analysis

We used descriptive statistics to analyze patient characteristics and collected data. Multivariate logistic regression analysis was used to determine the association between patient demographic and clinical characteristics and unintentional medication discrepancies. All analyses were performed using PSPP version 0.9.0 (Free Software Foundation, Inc. Boston, MA, USA), a free software for statistical computing and graphics (28). Study results were considered significant at *P* < 0.05.

## Results

### Study sample

Of 8726 patients admitted to the Clinical Department of Internal Medicine during the study period, 423 (4.8%) patients were randomly selected. Among the selected patients, 12 of 423 were excluded as they refused to provide their informed consent. Thus, the final study population included 411 patients. The mean age of the participants was 65 years (19-92 years) with around 50% of male participants (Table 1). Participants used six home prescription medications on average and had around five comorbidities.

**Table 1:** Patient baseline demographics and clinical characteristics (N = 411)

Characteristic	No. (%) of patients
Mean age (range, years)	65 (19-92)
≥65	211 (51.3)
46-64	161 (39.2)
<45	39 (9.5)
Sex	
male	220 (53.5)
Educational level	
no school or elementary school	134 (32.6)
high school	215 (52.3)
undergraduate	62 (15.1)
Residence	
living alone	59 (14.4)
living with family/caregiver	346 (84.2)
nursing home	6 (1.4)
Admission to hospital	
emergency	273 (66.4)
elective	138 (33.6)
Mean number of comorbidities (range)	4 (0-19)
Mean hospital length of stay (range, days)	9 (2-32)
Mean number of regular prescription home medications (BPMH*) (range)	6 (1-19)
The most common drug classes in BPMH (ATC† groups)	
A alimentary tract and metabolism	545 (20.7)
B blood and blood forming organs	243 (9.2)
C cardiovascular system	1065 (40.5)
M musculo-skeletal system	130 (4.9)
N nervous system	315 (12.0)
R respiratory system	127 (4.8)

*Best Possible Medication History (BPMH).

†Anatomical Therapeutic Chemical (ATC) drug classification system.

### Sources of information for BPMH

Interviews on medicine use were conducted by two study pharmacists and completed for all 411 participants. Information provided by the standardized interview was compared with other sources of information, which included previous medical records (79.3%), examination of medication vials (38.7%), communication with a GP (38.7%), patient's own medication list (8%), family/caregiver interview (6.6%), and communication with a community pharmacy (0.2%). Pharmacists assessed a mean of 2.7 information sources per patient to obtain the accurate and complete BPMH. The mean time for the completion of pharmacists’ chart reviews, patient/caregiver interviews, and filling out BPMHs was 20 minutes per patient.

### Unintentional medication discrepancies

Overall, 1200 cases of medication discrepancies were identified, with 998 (83.2%) of them being intentional and 202 (16.8%) unintentional. The overall rate of unintentional medication discrepancies was 0.48 per patient; 148 (35%) patients had one or more unintentional medication discrepancies. Twenty-six percent of all study participants had one, 7.1% had two, and 2.9% had three or more unintentional medication discrepancies. The most frequent type of unintentional medication discrepancy was an omission of essential therapy followed by an incorrect dose (Table 2). According to the potential severity of unintentional medication discrepancies, around 60% were assessed as having the potential to cause moderate or severe discomfort or clinical deterioration (Table 2). Discrepancies with the potential to cause severe discomfort or clinical deterioration are detailed in Table 3. The three most frequent drug classes involved in unintentional discrepancies according to the ATC classification included cardiovascular system, nervous system, and gastrointestinal system (Table 4). In addition, medicines used in the treatment of benign prostatic hypertrophy and chronic gout were often omitted.

**Table 2:** Types and potential severity of unintentional medication discrepancies in patients

Type of medication discrepancy	Number (%) of unintentional medication discrepancies	Potential severity of unintentional medication discrepancies (n,%)		
		class 1^*^	class 2^†^	class3^‡^
Drug omission	129 (63.9)	43 (33.3)	53 (41.1)	33 (25.6)
Incorrect dose	49 (24.2)	32 (65.3)	13 (26.5)	4 (8.2)
Drug commission	17 (8.4)	2 (11.8)	6 (35.3)	9 (52.9)
Incorrect frequency	4 (2.0)	3 (75.0)	1 (25.0)	0 (0)
Drug substitution	3 (1.5)	1 (33.3)	1 (33.3)	1 (33.3)
Incorrect route	0	0 (0)	0 (0)	0 (0)
**Total**	**202**	**81 (40.1)**	**74 (36.6)**	**47 (23.3)**

*Discrepancy had no potential to result in discomfort or clinical deterioration.

†Discrepancy had the potential to result in moderate discomfort or clinical deterioration.

‡Discrepancy had the potential to result in severe discomfort or clinical deterioration.

**Table 3:** Examples of unintentional medication discrepancies with the potential to cause severe discomfort or clinical deterioration in patients (Class 3)

	Medication discrepancy	
Reason for hospital admission	type	description
Polypus colonis	Drug omission	Patient reported use of warfarin 1.5 mg/d before admission, which was not ordered on admission.
Pancreatic duct stones	Drug addition	Patient was taking tramadol p.o. 100 mg/d at home. After being admitted to the hospital, without the physician’s knowledge, the patient continued to use tramadol from his own supply while being treated with tramadol s.c. 50 mg/d.
Abdominal pain	Drug addition	Amoxicillin and clavulanate was initiated although the patient reported allergy to amoxicillin.
Atrial fibrillation	Drug dose	Theophylline, 400 mg twice daily, was initiated in a patient older than 65 years.
Pyrexia	Drug substitution	Osveral 500 mg (Deferasirox) was ordered on admission, based on physician's assumption, instead of Osvaren 435 mg/235 mg (calcium acetate and magnesium carbonate).
Hypertension	Drug dose	Amlodipine, 20 mg/d, was ordered at admission (exceeding the maximum dose). Patient reported use of amlodipine 10 mg/d.
Rectal bleeding	Drug addition	Clarithromycin 500 mg was initiated without any indication.
Lung cancer	Drug addition	Ipratropium was initiated in therapy already containing tiotropium.

**Table 4:** The most frequent medication groups and their therapeutic subgroups susceptible to unintentional medication discrepancy, presented according to the Anatomical Therapeutic Chemical (ATC) classification

Anatomical Therapeutic Chemical (ATC) classification code	Number (%) of unintentional medication discrepancies	
C (Cardiovascular system)	51 (25.2)
C01 Cardiac therapy	6
C02 Antihypertensive	3
C03 Diuretics	7
C07 Beta blocking agents	8
C08 Calcium channel blockers	4
C09 Agents acting on the renin-angiotensin system	11
C10 Lipids modifying agents	12
N (Nervous system)	47 (23.3)
N02 Analgesics	8
N03 Antiepileptics	1
N04 Anti-Parkinson drugs	3
N05 Psycholeptics	30
N06 Psychoanaleptics	5
A (Alimentary tract and metabolism)	46 (22.8)
A02 Drugs for acid related disorders	22
A05 Bile and liver therapy	2
A07 Antidiarrheals, intestinal anti-inflammatory/anti-infective agents	3
A10 Drugs used in diabetes	6
A11 Vitamins	6
A12 Mineral supplements	7

### Association between patient characteristics and unintentional medication discrepancies

The association between patient characteristics and the likelihood of unintentional medication discrepancy was assessed in a multivariable model (Table 5). No significant associations were found for the age, sex, educational level, residence, type of hospital admission (elective vs emergency admission), outpatient visits in the prior year, previously experienced adverse drug events, number of comorbidities, and length of hospital stay. Increased number of preadmission medications (OR 1.19; 95% CI, 1.10-1.29, *P* < 0.001) was found to be the strongest predictor of unintentional medication discrepancies. Each additional medication increased the odds for the patient experiencing at least one medication error by 19%. Low level of patients’ understanding of preadmission medications was significantly associated with unintentional medication discrepancies (OR 1.79, 95% CI, 1.01-3.16, *P* = 0.046), although the odds ratio confidence interval was rather wide (1.01-3.16).

**Table 5:** risk factors for unintentional medication discrepancies

Patient characteristic	*P*-value^*^	Odds ratio (95% confidence interval)
Age	0.510	1.01 (0.99-1.02)
Sex		
male	0.782	0.94 (0.60-1.47)
female		Ref
Education level		
no school or elementary school	0.507	1.30 (0.60-1.47)
high school	0.789	1.10 (0.53-2.29)
community college	0.901	1.09 (0.27-4.47)
undergraduate		Ref
Type of residence		
living alone	0.478	0.49 (0.07-3.48)
living with family, caregiver	0.206	0.29 (0.04-1.96)
nursing home		Ref
Admission		
unplanned	0.185	0.71 (0.43-1.18)
planned		Ref
Patient’s understanding of preadmission medications		
low	0.046	1.79 (1.01-3.16)
medium	0.417	1.26 (0.72-2.22)
high		Ref
History of ADEs^†^	0.800	1.06 (0.67-1.67)
Recent hospitalization	0.366	0.81 (0.51-1.28)
Number of comorbidities	0.211	0.95 (0.87-1.03)
Length of hospital stay	0.223	1.03 (0.98-1.07)
Number of medications (BPMH^‡^)	<0.001	1.19 (1.10-1.29)

*Multiple logistic regression.

†Adverse Drug Event.

‡Best Possible Medication History.

## Discussion

### Summary key findings and comparison with published studies

Clinical pharmacists in our study detected a high number of medication discrepancies, many of which were assessed as having the potential to cause severe discomfort or clinical deterioration in patients. These results confirmed that clinical pharmacists are important in both detection and prevention of medication errors and correspond to the findings of previous studies on pharmacists' role in medication reconciliation (6-8,12,13) and a recent systematic review showing that pharmacist-led medication reconciliation programs are a promising strategy for safe patient transitions (19). In addition, our study provided the first evidence on the discontinuity of care at the point of hospital admission and is the first to assess its impact on patient care process in a clinical setting in Croatia. Moreover, to the best of our knowledge, this study is among the first studies conducted in South-Eastern European countries to have evaluated the incidence and potential clinical impact of unintentional medication discrepancies, thus contributing to the evidence base regarding the safety of seamless pharmaceutical patient care (29).

The incidence of unintentional medication discrepancies in the published literature varies considerably depending on the hospital setting including surgery, internal medicine, geriatric or emergency departments (9,12,13,30). A higher proportion reported in the published data (3-5) could partially be explained by a less rigorous methodology, ie, the use of outcome measures not differentiating between medication history-taking errors and reconciliation errors, or simply by the fact that more unintentional medication discrepancies were present and consequently captured in those study samples. In our study, only unintentional medication discrepancies confirmed by hospital physicians were considered to be medication errors. Our results are comparable to the findings of studies performed on general medical wards and those that used a methodology similar to ours. Gleason et al found over one-third of patients having a medication error at admission (6), while Quélennec et al (8) demonstrated similar results with a third of patients having one or more unintentional medication discrepancies in a sample of 256 general medicine inpatients.

The most common type of unintentional medication discrepancy in our study was the omission of pre-admission regular therapy, followed by an incorrect dose, which is similar to previous research findings (6,8,30,31). Majority of unintentional medication discrepancies were found for cardiovascular agents and drugs acting on nervous and gastrointestinal system, which is consistent with findings of other studies (5,6,8). Moreover, psycholeptic drugs (N05), namely benzodiazepines, were the most frequent therapeutic subgroup associated with unintentional medication discrepancies. They were often omitted and abruptly discontinued, possibly leading to withdrawal adverse effects, or needlessly introduced during hospital stay. Medications to treat patients' comorbidities, such as gout and functional symptoms of benign prostatic hyperplasia, were often overlooked, most probably due to polypharmacy in these patients and/or the complexity of the remaining medication regimen.

Each unintentional medication discrepancy was assessed for its potential harmfulness and severity. In our study, over half of the identified unintentional medication discrepancies had the potential to cause moderate to severe discomfort or clinical deterioration (Classes 2 and 3). The incidence of unintentional, potentially harmful medication discrepancies found in the literature varies substantially (3,6,10,32,33). For the assessment of potential clinical severity, different classification methodologies are in use. Adjusted National Coordinating Council for Medication Error Reporting and Prevention Index (NCC MERP Index) synthesizing errors into three categories was used in previous studies (6,8). Similarly, methodology used in our study also classified medication errors in three categories where the first class had no potential to cause harm. However, the standard for evaluating the potential severity of unintentional medication discrepancies has not yet been established and it is, therefore, not possible to make a valid comparison between various studies.

### Clinical pharmacists-led medication reconciliation and other solutions

We found that around 60% of clinically significant medication discrepancies had the potential to threaten patient safety. It is a considerable incidence, which should be detected and prevented. Therefore, the role of clinical pharmacists in identifying medication discrepancies is of notable importance and considerable efforts need to be taken to strengthen their position in a medication reconciliation process and health care system in general. Practices in other countries have clearly demonstrated that it is feasible, time-saving, and cost-effective to include other health care providers, such as pharmacy technicians, pharmacy or medical students, in a medication reconciliation process (14,34). It increases the time available to the pharmacist for resolving drug therapy problems and allows them to provide more in-depth clinical services (35). Furthermore, in the Netherlands, pharmacy technicians play an important role in obtaining BPMH after being trained and under the supervision of hospital pharmacists. Studies conducted in Dutch hospitals demonstrated that pharmacy technicians could be successfully assigned to medication reconciliation process, because BPMH obtained by pharmacy technicians was comparable to that obtained by pharmacists when examining the mean number of discrepancies per patient (14,36). However, in addition to obtaining BPMH, medication reconciliation process used in our study included intervening and rectifying medication errors by consultation with physicians when needed. It required a more complex skill mix from involved health care professionals, ie, clinical pharmacists. Moreover, our goal was to empower pharmacists, accentuate their unique role as medicine experts, and finally employ them as accessible workforce in the hospital. In addition, a position of pharmacy technicians in Croatia is unsatisfactory due to workload shortages. Their number is still undersized and their competencies are neither recognized nor defined.

Pharmacists are the only health care professionals who do not have direct patient care responsibilities. Although clinical pharmacy services are delivered in inpatient environment and the pharmacist works “closely” with the physician, it is the physician or the nurse who still assumes ultimate responsibility for most decisions or recommendations made by the pharmacist. Therefore, in an attempt to start applying professional patient care practice in the same manner and according to the standards required from other health professionals, the advantage was given to pharmacists rather than pharmacy technicians or nurses.

Other potential solutions for increasing patient safety include electronic medication reconciliation tools (11). Previous research explored the effect of an electronic medication reconciliation application combined with process redesign on decreasing medication discrepancies with potential for harm. The study results revealed a significant reduction in unintentional medication discrepancies with potential for harm (11). Still, there were problems associated with incomplete and inaccurate electronic sources of ambulatory medications and the fact that many hospitals had not developed computerized provider order entry (CPOE) needed for computerized medication reconciliation.

Patients should be encouraged to play a more active role during care transitions, when serious quality deficiencies happen. Patients and their family caregivers, being the main source of information, should be more actively involved through medication self-management, patient-centered record, follow-up and red flag interventions, to improve quality and safety during care transitions. Previous evidence demonstrated that prompting patients to assume a more active role reduced hospitalization rates (37). In this study, pharmacists completed interview with all included patients or their caregivers and performed medication reconciliation in a thorough and extensive manner, while enabling them to assume a more active role by providing patients with various tools. Based on the level of patients’ understanding of their treatment and their adherence, they were educated properly about medications and the importance of taking medications regularly.

### Risk factors for unintentional medication discrepancies

We found two predictors of unintentional medication discrepancies: an increased number of drugs and a low level of patients’ understanding of their home medications. Previously published studies have already confirmed the association between these two predictors and the occurrence of unintentional medication discrepancies (6,7,30). Therefore, pharmacists should be focused on polypharmacy and a low level of therapy regimen understanding.

### Study limitations

Our study had several limitations. First, it was conducted in a single Croatian hospital and the results may not be generalizable to other settings. Second, the rating method for potential severity of medication discrepancies has not been validated. However, methodology used in our study was strengthened by the fact that clinical pharmacists conducted the medication reconciliation process. The literature confirms that pharmacists identify more medications per patient than other health care professionals thus providing more accurate and complete medication histories (16,18). Consequently, more clinically relevant medication discrepancies of higher impact are identified through pharmacy-led medication reconciliation (19). Furthermore, pharmacists used a wide variety of sources of information and the data collection was very extensive, with a large number of demographic and clinical characteristics.

In conclusion, clinical pharmacist-led medication reconciliation implementation program revealed a high rate of medication errors in patients at hospital admission, with more than a half of them having the potential to cause moderate to severe discomfort or clinical deterioration, thus threaten patient safety. These results confirm that clinical pharmacists play an important and invaluable role in detection of discrepancies and prevention of adverse patients’ outcomes. Other hospitals can use the same methodology and implement clinical pharmacist-led medication reconciliation at hospital admission. Since an increased number of drugs and a low level of patients’ understanding of preadmission therapy were found as predictors of medication errors, targeted patient education provided by pharmacists could render a feasible and sustainable solution.
